# p53 signaling modulation of cell cycle arrest and viral replication in porcine circovirus type 2 infection cells

**DOI:** 10.1186/s13567-016-0403-4

**Published:** 2016-11-29

**Authors:** Dan Xu, Qian Du, Cong Han, Zengguo Wang, Xiujuan Zhang, Tongtong Wang, Xiaomin Zhao, Yong Huang, Dewen Tong

**Affiliations:** College of Veterinary Medicine, Northwest A&F University, 22 Xinong Rd, Yangling, Shaanxi 712100 People’s Republic of China

## Abstract

Porcine circovirus type 2 (PCV2) is a ubiquitous pathogen in the swine industry worldwide. Previous studies have shown that PCV2 infection induces host cell apoptosis through up-regulation of p53. To further identify the regulatory roles of p53 signaling in the process of PCV2 infection, we established *p53* gene knockout PK15 cell lines using the genomic editor tool CRISPR/Cas9, and further investigated the roles of p53 in modulating the cell cycle and viral replication in this study. The results show that PCV2 infection induced obvious S phase accumulation in wild-type PK15 cells and a compromised S phase accumulation in the *p53* gene mutation cells (813PK15^*p53m/m*^), but did not induce obvious S phase accumulation in the *p53* gene knockout cells (148PK15^*p53*−*/*−^) compared with the respective mock infection. PCV2 infection activated p53 signaling, up-regulated the expression of p21, Cyclin E, and down-regulated Cyclin A, CDK2. In p53 deficient cells, however, PCV2-induced changes in Cyclin A, CDK2, and Cyclin E were efficiently reversed to the basal levels. Detection of PCV2 replication showed decreased viral ORF1 genomic DNA in p53 deficient cells (148PK15^*p533*−*/*−^) and p53 mutated cells (813PK15^*p53m/m*^) compared with p53 wild-type cells after different synchronization treatment. Furthermore, PCV2 viral genomic DNA and Cap protein levels were higher in the cells released from S phase synchronized cells than in the cells released from the G0/G1 phase or G2/M phase-synchronized, or asynchronous cells after 18 h post-infection. Taken together, this study demonstrates that PCV2 infection induces S phase accumulation to favor viral replication in host cells through activation of the p53 pathway.

## Introduction

PCV2, belonging to the family Circoviridae, is the main pathogen to cause porcine circovirus associated diseases (PCVAD) [1], posing a huge threat for world pig husbandry [2]. As a tiny DNA virus, PCV2 infection requires host cells to provide necessary resources for replication themselves, thus disturbing a variety of cell signaling pathways to modulate the host cell cycle, proliferation, survival and death to facilitate their infection and replication [3, 4]. Among the signaling pathways, p53 signaling is essential for control of quiescent cell entry into the cell cycle, and regulating cellular DNA replication [5]. However, the roles of p53 signaling in modulating cell cycle and PCV2 replication has not been defined up to date.

Numbers of studies have broadened our understanding of the roles of p53 signaling in the process of different virus infection and replication. For instance, Kaposi’s sarcoma herpesvirus (KSHV) activates host p53 signal and induces G2 phase arrest to promote the onset of virus replication [6]. Prototype foamy virus (PFV) promotes p53 level increase by knockdown of Pirh2 to contribute to the latency of PFV infection [7]. Herpes simplex virus type 2 infection can phosphorylate p53 protein to induce the G0/G1 phase arrest [8]. PRRSV manipulates the host factors mdm2 and p53 via its Nsp1α to increase viral replication at the early stage of infection [9]. Indeed, previous studies have shown that PCV2 ORF3 protein specifically interacts with porcine ubiquitin E3 ligase Pirh2 to promote p53 accumulation [10], playing an important role in PCV2 pathogenesis [11], which indicates the key role of p53 in the interaction of PCV2 and the host. However, in-depth study of the roles of p53 signaling in the process of PCV2 was limited due to lacking of *p53* deficient cell line in porcines.

In this study, with the help of the CRISPR/Cas9 system, we constructed p53 deficient and mutant porcine cell lines, and further detected and compared the difference of cell cycle profiles and viral replication between the p53 wild-type, p53 deficient and p53 mutant porcine cell lines. This study allows us to deeply explore and confirm the roles of p53 signaling in modulating cell cycle and PCV2 replication.

## Materials and methods

### Cells, virus and antibodies

Porcine kidney 15 (PK15) cells purchased from ATCC (CCL-33) were cultured in Dulbecco’s Modified Eagle’s Medium (Gibco BRL, Gaithersburg, MD, USA) supplemented with 10% heat-inactivated fetal bovine serum (Thermo Scientific HyClone, Beijing, China), and incubated at 37 °C in a 5% CO_2_ atmosphere incubator. The PCV2 strains (GenBank No. EU366323) used in this study were isolated and purified previously by our team and stocked in our laboratory, the UV-inactivation was performed by UV radiation of the virus for 45 min in the hood. The anti-PCV2 Cap primary antibodies were produced by our team [12, 13]. The primary monoclonal rabbit antibodies of p53, p21 and anti-BrdU were purchased from Cell Signaling (Cell Signaling Technology, Danvers, MA, USA). CDK2, Cyclin A and Cyclin E antibodies were purchased from Santa Cruz Biotechnology (Santa Cruz, California, CA, USA). The monoclonal antibody of β-actin was purchased from sigma (Sigma-Aldrich, St. Louis, MO, USA). The FITC goat anti-mouse IgG was purchased from BD Biosciences (BD, San Jose, CA, USA).

### Cell cycle analysis

The ratio of cells in each phase of the cell cycle was determined by DNA content using propidium iodide (PI) staining followed by flow cytometric analysis. The cells plated at a density of 1 × 10^6^ cells/flask were treated with the indicated Multiplicity of infection (MOI) of PCV2 for the indicated times as described in the figure legends. The cells were trypsinized, washed twice with PBS, and fixed with 70% ice-cold ethanol at −20 °C overnight. Fixed cells were washed with cold PBS and resuspended with PI staining solution containing 50 mg/mL PI (Sigma-Aldrich), 100 mg/mL RNase A (TIANGEN Biotech, Beijing, China), and incubated in the dark for 30 min. The samples were analyzed using a flow cytometer (Accuri™ C6, BD Biosciences, San Diego, CA, USA).

### CRISPR/cas9 KO cell

Targeting sites in the *p53* gene were selected using the CRISPR program (Genome Engineering. Broad Institute Cambridge, MA, USA) Oligonucleotide pairs for the target sequences were annealed and the resulting fragments were then cloned into the BsmB I sites of lentiCRISPRv2 plasmid (Addgene), and co-transfected into HEK293T cells with the packaging plasmids psPAX2 (AddGene 12260) to generate the lentivirus. 72 h after the transfection, the supernatant was collected after three cycles of frozen-thawed. Titers of the obtained lentivirus expressing the target sequences were determined by qPCR. Finally, the CRISPR/Cas9 mediated P53 knockout cells were selected from lentivirus infected PK15 cell lines that were cultured in puromycin (500 ng/mL) DMEM medium for at least 14 days. Genomic DNA sequence from PK15 cells was determined using primers: 148-F: 5′-GACTCCTGTTGTTCCCATCCA-3′; 148-R: 5′-AGGGAGCCAGCAGTCAAATG-3′; 813-F: 5′-GGGACGGAACAGCTTTGAGGT-3′; 813-R: 5′-CTGTTGGCAAATGCCCCAAA-3′.

### Cell synchronization

Cells synchronized in G1/G0 phase were obtained by serum starvation. PK-15 cells were cultured in serum-free medium for 24 h or 48 h, and then cells were washed with PBS and plated in fresh media to start PCV2 incubation for 1 h and cultured in 2% FBS DMEM medium for 18 or 24 h for later analysis. Double thymidine block was used for early S phase synchronization. The cells were treated for 12 h with 2 mM thymidine, after which cells were washed and released into fresh media with MOI = 1 PCV2 virus then incubated for 1 h, and cultured in 2% FBS DMEM medium for 18 h. The cells were treated with 100 ng/mL nocodazole for 16 h until arrest at the G2/M phase, then the cells were released by washing with PBS and plated in fresh media to start PCV2 incubation for 1 h and culture in 2% FBS DMEM medium for 18 h for later analysis.

### Detection of virus replication

The cells were seeded in culture plates at a density of 1 × 10^6^ cells/well, and cultured to reach approximately 60–70% confluence. PCV2 strains were used to infect the cells at a multiplicity of infection of 1. Viral ORF1 fragments were determined in each of the PK15 cell lines using primer PCV-F: 5′-AGTACCGGGAGTGGTAGGAG-3′; PCV-R: 5′-GTTGAATTCTGGCCCTGCTC-3′. The supernatant and the attached cells were collected together to extract the DNA.

### BrdU incorporation assay

For labeling of S-phase cells, BrdU was added in mid-log phase cells at a final concentration of 10 µM and incubated for 1 h at 37 °C. The cells were harvested and washed with PBS + 1% BSA. The cells were further resuspended and fixed overnight in chilled 70% ethanol at a cell density of 2 × 10^6^ cells/mL at 4 °C. Further, ethanol was removed and the cells were incubated in 2 N HCL+ 0.5% Triton X-100 solution for 30 min at RT followed by washing in 0.1 M Sodium tetraborate solution for 2 min. Then the cells were resuspended in PBS/0.5% Tween-20 + 1% BSA and incubated with anti-BrdU antibody for 1 h at RT. The cells were washed again with the same buffer and incubated in PBS/0.5% Tween-20 + 1% BSA with FITC goat antimouse antibody for 0.5 h at RT. Then the cells were washed and resuspended in RNase + PI at RT for 0.5 h min and finally centrifuged. The cells were collected for flow cytometer analysis.

### Western blot

The protein expression was measured by western blot as described previously [14]. Briefly, PK15 cells were cultured in a flask, infection was as indicated at an MOI of PCV2 virus and harvested at an 18 h interval as described above. Whole-cell protein extract was prepared as described above. The protein extract from each group was loaded to 12% SDS-PAGE, and transferred to PVDF membranes. After blocking for 1 h at room temperature, the membranes were incubated with primary antibody at 4 °C overnight. After being incubated for 1.5 h with HRP-conjugated IgG second monoclonal antibody at room temperature, the membranes were immersed in an ECL reagent (Pierce, Rorkford, IL, USA) and visualized in X-ray films. The optical density of each band was quantified using ImageJ analysis software (NIH, NY, USA) with β-actin as an internal control.

### Statistical analyses

All data were presented as “mean ± SEM” in triplicate, and analyzed with the Student’s *t* test using SPSS 17.0 software (SPSS Inc., Chicago, IL, USA). Differences with the controls were considered significant when *p* < 0.05.

## Results

### PCV2-infected cells accumulated at the S phase of the cell cycle

**Figure 1:**
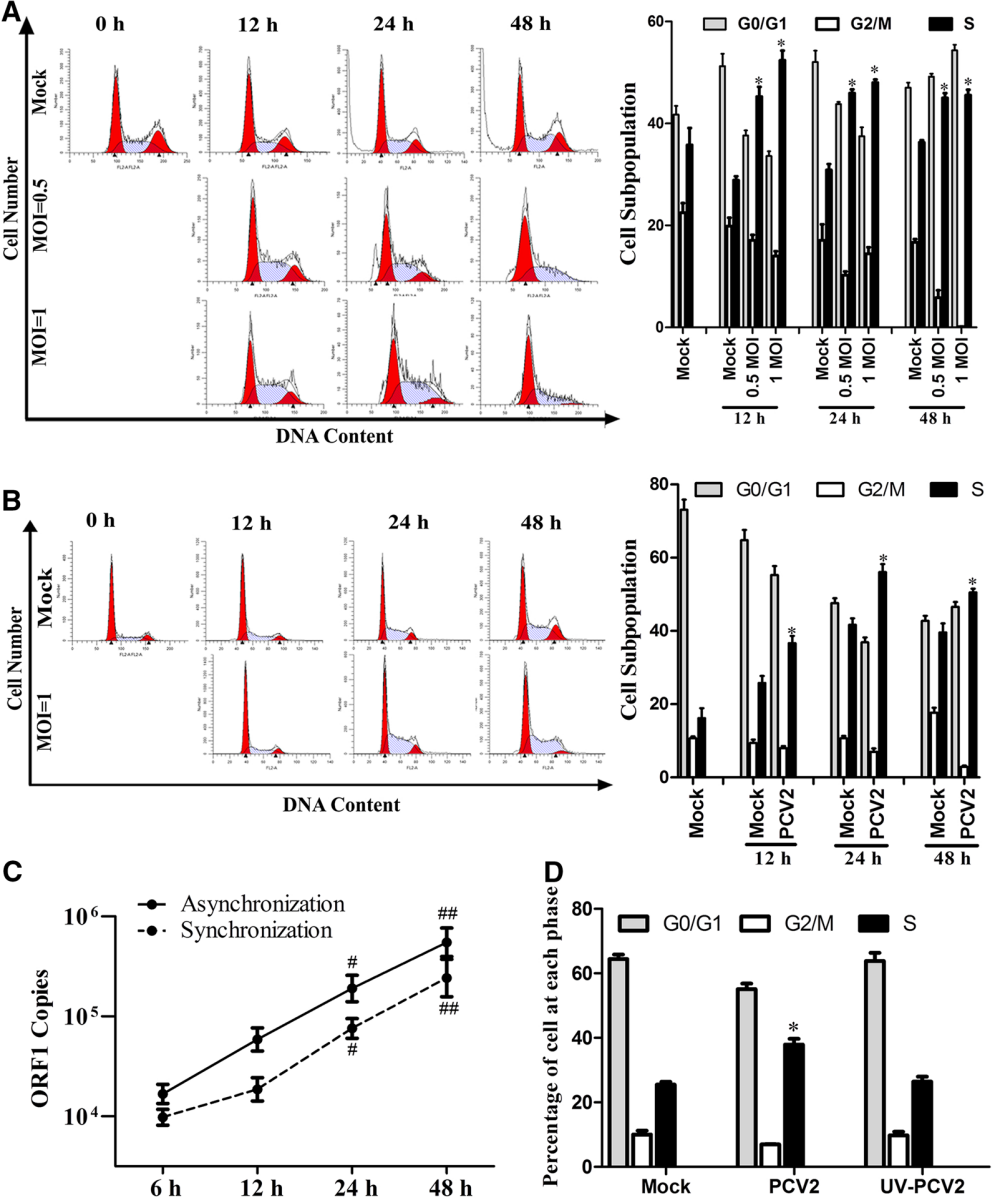
**Cell cycle distribution of PCV2-infected PK15 cells. A** Flow cytometry analysis of cell cycle subversion in mock-infected or PCV2-infected PK15 asynchronously growing cells. Cells were fixed in ethanol at 12, 24, and 48 h post-infection (pi) with 0.5 and 1 MOI of PCV2, DNA was stained with propidium iodide and applied to flow cytometry. **B** Flow cytometric analysis of cell cycle profiles in serum-starved PK15 cells with mock or 1 MOI of PCV2 infection. Cells were fixed in ethanol at the indicated time and stained with PI. The percentages of each cell cycle phase (G0/G1, G2, S) were output and analyzed by ModFit LT software in the right panel. **C** qPCR determined the level of PCV2 ORF1 genomic DNA at the indicated infection time in PCV2-infected asynchronic and G0/G1 synchronic cells. **D** Cell cycle profiles were measured after 1 MOI PCV2 or UV-inactivated PCV2 infected cells for 24 h. The data are mean ± SEM of three independent experiments. *p < 0.05, versus mock infected cells at the same time points (**A**, **B**, **D**). ^#^ *p* < 0.05, ^##^ *p* < 0.01 versus 6 h pi, in the same infected cells (**C**).

Interference of cell cycle progression is a common response for many virus infections [15]. In order to investigate the influence of PCV2 infection on host cell cycle progression, PCV2 infected asynchronously PK15 cells for different times at different MOI indicated, and cell cycle progression was analyzed by flow cytometry. The S phase accumulation was observed at 12 h post-infection (pi), and further increased at 24 and 48 h pi in PCV2-infected cells (Figure 1A). At 24 h pi, the cell population of S phase in PCV2-infected cells was close to 1.5-fold higher than that in mock-infected cells. At 48 h pi, the proportion of cells in the S phase remained at higher levels in PCV2-infected cells compared to mock infection cells, and the S phase cell accumulation reached a relatively stationary level in different MOI of PCV2 infected cells. These results indicate that PCV2 infection interferes with the cell cycle progression by delaying (prolonging) the S phase of the cell cycle.

We further addressed serum-starved quiescence cells to monitor the quantification of DNA cycle fractions upon PCV2 infection. PK15 cells were serum-starved for 24 h followed by PCV2 infection. S phase cell populations increased by 1.32-, 1.46- and 1.31-fold at 12, 24 and 48 h pi, respectively, in PCV2-infected cells, compared to that in mock infection cells, which was accompanied by a concomitant decrease in the G0/G1 or G2/M phase cell populations (Figure 1B), indicating that PCV2 blocked the cell cycle at the S phase. At 48 h pi, the G2/M population of the cell was markedly reduced, suggesting the complete blockage of the S-G2 transition in PCV2-infected cells. Meanwhile, viral ORF1 genomic DNA in PCV2-infected cells (MOI = 1) was monitored by qPCR from the asynchronic and synchronic of infection cells, the total ORF1 genomic DNA were amplified from 12 h, and significantly increased at 24 and 48 h pi (Figure 1C). To determine whether PCV2-induced S phase accumulation was dependent on the viral replication, 1 MOI of UV-inactivated PCV2 was inoculated with synchronized PK-15 for 24 h to measure the cell cycle profiles by flow cytometry. The results show that there was no significant difference for cell cycle distribution between the UV-inactivated PCV2 group and the mock infection group (Figure 1D), suggesting that UV-inactive PCV2 virus did not alter cell cycle distribution, and viral replication was required for induction of cell cycle arrest in PCV2-infected cells. These results indicate that PCV2 infection induces numerous host cells to accumulate in the S phase, which might be beneficial for virus replication.

### Loss of p53 impairs PCV2 induced S phase accumulation and viral propagation

**Figure 2:**
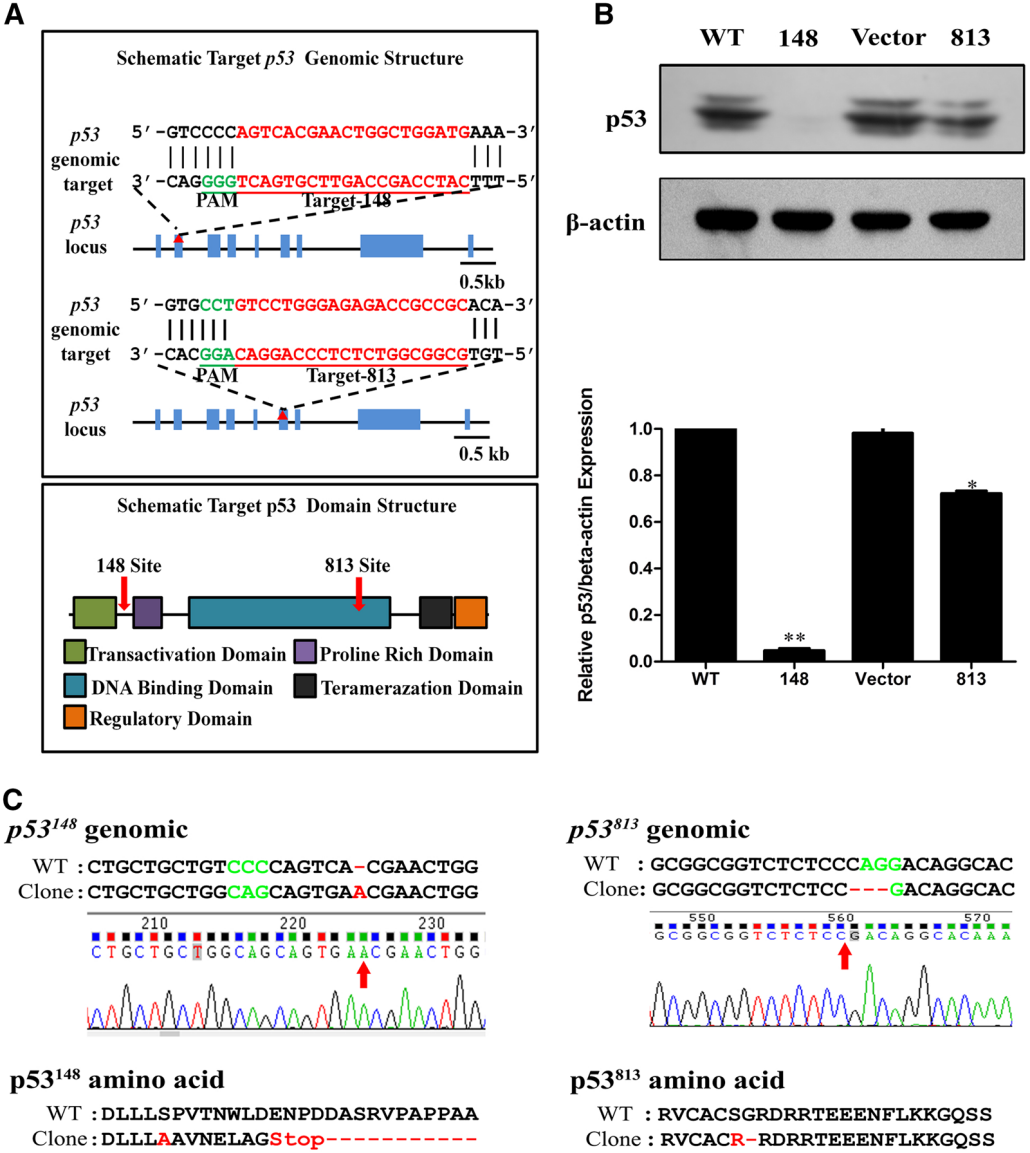
**Construction of p53 knock-out PK15 cells using CRISPR/Cas9 genome editing system. A** Schematic chromatogram representation of sgRNA targeting at the *p53* genomic region. PAM sequences are underlined and highlighted in green. sgRNA targeting sites are highlighted in red. The red arrow indicates a putative cleavage site. The lower panel represents the functional domain of the *p53* gene. The selected DNA oligos for the gRNA were annealed and cloned into the lentivirus vector for the CRISPR/Cas9 system. **B** Western blot analysis of the p53 expression after CRISPR/Cas9 lentivirus system targeting *p53* locus infected PK15. The cells infected with lentiviruses containing gRNA-148 (148), gRNA-813 (813) or control gRNA (vector) were selected by puromycin, then puromycin-resistant cells and wild-type PK15 cells (WT) were analyzed using western blot. The data are mean ± SEM of three independent experiments. *p < 0.05, **p < 0.01 versus WT cells. **C** Sequencing of *p53* locus amplified from the 148-gRNA (*p53* ^148^) and gRNA-813 (*p53* ^813^) generated cell clone. Red arrows indicate insertions or mutations. PAM sequences are highlighted in green. Red dashes indicate deleted bases or amino acids. Red characters indicate inserted base or mutated amino acids.

In order to determine the roles of p53 in PCV2 infection and S phase accumulation, the *p53* gene was mutated using the CRISPR/Cas9 system to address its role in PCV2–cell interaction. gRNA for mutagenesis of the *p53* gene were designed according to the CRISPR/cas9 program. Target sites were selected from sequences with low homologies to other genome sites to avoid off-target mutagenesis. gRNA148 targeted around the N-terminal domain of p53 which is related to the transactivation subdomain and proline-rich fragment; gRNA 813 targeted the middle area of the central core region, which encodes the DNA binding domain necessary for protein–DNA interaction (Figure 2A). Puromycin-resistant PK15 cells were selected at 500 ng/mL and further amplified, then subsequently examined by western blot. The 148-gRNA generated clone showed complete elimination of the p53 protein, while the 813-gRNA generated clone slightly decreased expression as presented (Figure 2B). To further check the genomic changes of the two positive clones, we designed two pairs of primers flanking three gRNA target sites to perform PCR amplification and sequencing. The 148-gRNA driven cell line (148PK15^*P53*−*/*−^) genomic sequencing displayed a single nucleotide insertion leading to the serine 47 site mutation to alanine and introduced an early stop codon, while the 813-gRNA driven cell line (813PK15^*P53m/m*^) displayed mutations at serine 271 and glycine 272 sites into arginine (Figure 2C).

**Figure 3:**
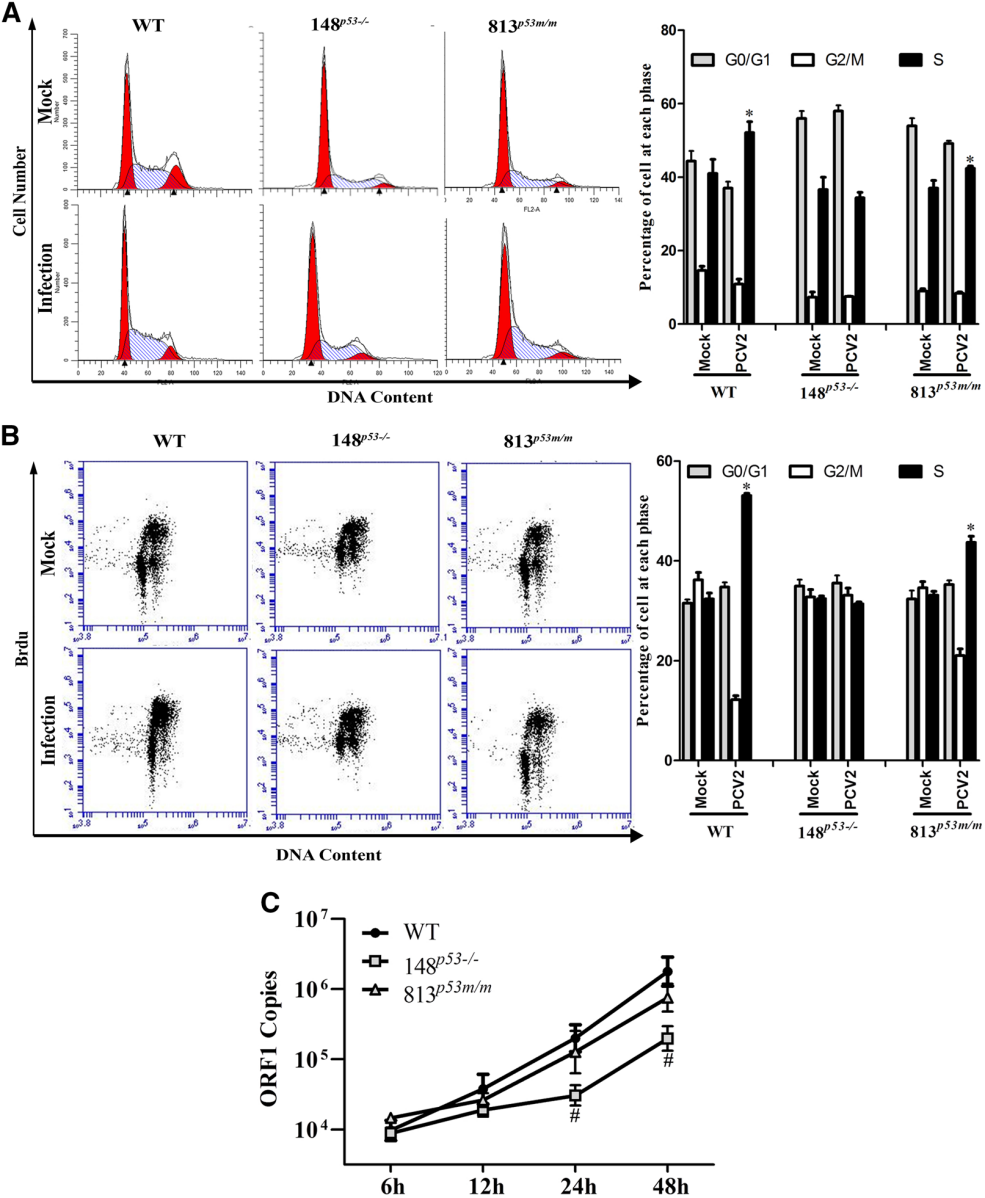
**p53 mediates PCV2-induced S phase accumulation and promotes viral multiplication.** **A** Flow cytometry analysis of cell cycle subversion in serum-starved WT PK15, 148PK15^*P53*−*/*−^, and 813PK15^*P53m/m*^ cells upon PCV2 infection (MOI = 1). Cells were fixed in ethanol at 24 h pi, DNA was stained with propidium iodide and applied to flow cytometry. The percentages of each cell cycle phase (G0/G1, G2, S) were output and analyzed by ModFit LT software in the right panel. **B** Quantitative analysis of S-phase entry (percentage of BrdU-positive cells) exposed to the 1 MOI PCV2 for 12 h and successively cultured in the BrdU for 1 h. **C** Absolute quantification of PCV2 replication in WT, 148PK15^*P53*−*/*−^, and 813PK15^*P53m/m*^ cells. 1 MOI of PCV2-infected asynchronously growing WT, 148PK15^*P53*−*/*−^, or 813PK15^*P53m/m*^, and viral ORF1 DNA were determined by the combination of the supernatant fluids and cytoplasmic extraction at 12, 24, 48 h pi. The data are mean ± SEM of three independent experiments. *p < 0.05 versus mock infection in the same kinds of cells. ^#^ *p* < 0.05 versus PCV2-infected WT cells at same time point.

Next, the p53 knockout PK15 cells (148PK15^*P53*−*/*−^) and p53 mutated PK15 cells (813PK15^*P53m/m*^), as well as the wild-type PK15 cells (WT), were infected with 1 MOI of PCV2 for 24 h, then flow cytometry analyzed cell cycle distribution. The results show that compared with PCV2-infected WT cells, the percentage of S phase cells significantly decreased in both PCV2-infected 148PK15^*P53*−*/*−^ cell and PCV2-infected 813PK15^*P53m/m*^ cells (Figure 3A). Consistently, PCV2 infection did not induce S phase accumulation in p53 knockout PK15 cells (148PK15^*P53*−*/*−^) compared with mock infection, while PCV2 infection slightly increased the percentages of S phase cells in 813PK15^*P53m/m*^ cells. To further determine the exact effects of p53 deficiency and mutation on the cell cycle distribution of PCV2-infected cells, we detected and compared the extent of S phase DNA synthesis between the PCV2-infected group and mock-infected group in wild-type PK15 cells, *p53* gene knockout cells (148PK15^*p53*−*/*−^) and *p53* gene mutation cells (813PK15^*p53m/m*^) by BrdU incorporation assay, which showed similar results as Figure 3A. Following PCV2 infection, the extent of S phase DNA synthesis increased from 33.0 to 51.3% in wild type cells, whereas the p53 knockout cells exhibited a relatively stabilized DNA synthesis rate after PCV2 infection even though the p53 knockout cell has an initial relatively tight cluster cell cycle phase distribution and DNA synthesis rate (Figure 3B). At 6 h pi, 148PK15^*P53*−*/*−^ cells, 813PK15^*P53m/m*^ cells and WT cells did not have significantly different levels of viral DNA. At 24 and 48 h pi, the 148PK15^*P53*−*/*−^ cells exhibited lower viral level than WT cells, while 813PK15^*P53m/m*^ cells just showed a slight reduction compared with WT cells in viral level (Figure 3C). These results demonstrate that p53 plays a pivotal role in PCV2-induced S phase cell accumulation and viral replication.

### p53 mediates up-regulation of p21 and Cyclin E and down-regulation of Cyclin A, CDK 2 in PCV2-infected cells

**Figure 4:**
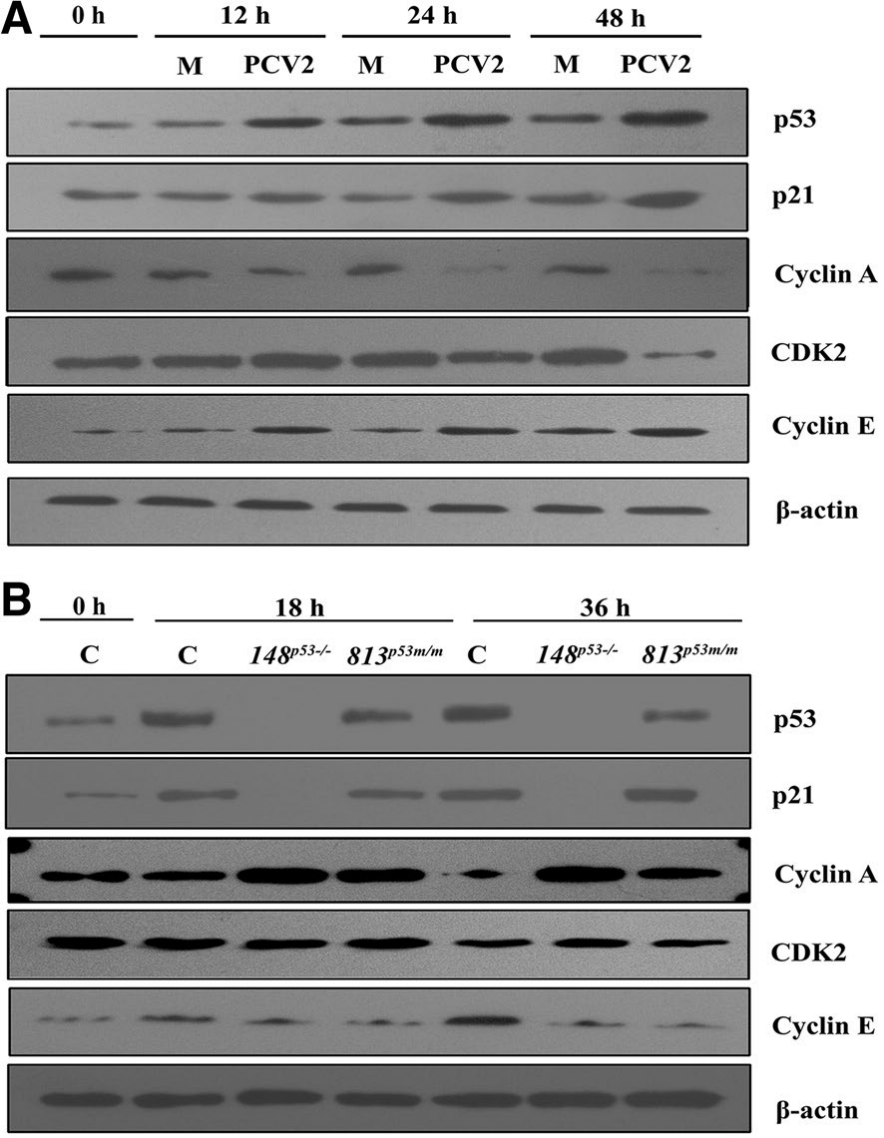
**p53 modulates cell cycle regulators in the process of PCV2 infection. A** Western blot analysis of the levels of cell cycle proteins in PCV2-infected PK15 cells. Cells were collected from Mock-infected or PCV2-infected (MOI = 1) at the indicated time points and processed for western blot analysis. **B** Cell cycle proteins change in WT, 148PK15^*P53*−*/*−^, and 813PK15^*P53m/m*^ cells with mock or PCV2 infection. Cells were collected at the indicated time points and subjected to western blot analysis as described in “Materials and methods” section. Data represent three independent experiments.

To elucidate the potential mechanisms involved when PCV2 infection induces S phase accumulation and p53 regulates cell cycle progression in infection cells, we examined the Cyclin and CDK proteins that are involved in the regulation of cell cycle transition. As shown in Figure 4A, a marked increase (about 2.3-fold) in p53 protein level was observed in WT PK15 cells 12 h after PCV2 infection, followed by p21 and Cyclin E up-regulation, whereas Cyclin A and CDK2 levels were significantly decreased in PCV2-infected cells at 24 and 48 h pi. The up-regulated expression level of Cyclin E may contribute to the cell cycle progression from G0/G1 to S phase. These results indicate that the increase of p53, p21 and Cyclin E together with the decrease of Cyclin A and CDK2, might be involved in the induction of the S phase accumulation in PCV2 infection cells.

However, in the *p53* knockout PK15 cells (148PK15^*P53*−*/*−^), p53 could not be detected, and p21 also could not be detected at 18 and 36 h after PCV2 infection. In accordance with these changes, the *p53* knockout cells exhibited relatively higher levels of Cyclin A and CDK2 and relatively lower levels of Cyclin E compared with wild-type cells at 18 and 36 h pi (Figure 4B). Meanwhile, p53 mutated PK15 cells (813PK15^*P53m/m*^) showed relatively lower levels of p53, Cyclin E and relatively higher levels of Cyclin A when compared with WT cells, but relatively lower levels of Cyclin A and CDK2 when compared with 148PK15^*P53*−*/*−^ cells at 18 and 36 h pi. These results suggest that PCV2 induced S phase accumulation through p53 mediated up-regulation of p21, Cyclin E and down-regulation of Cyclin A, CDK 2 proteins in PK15 cells.

### S phase accumulation promotes PCV2 replication

**Figure 5:**
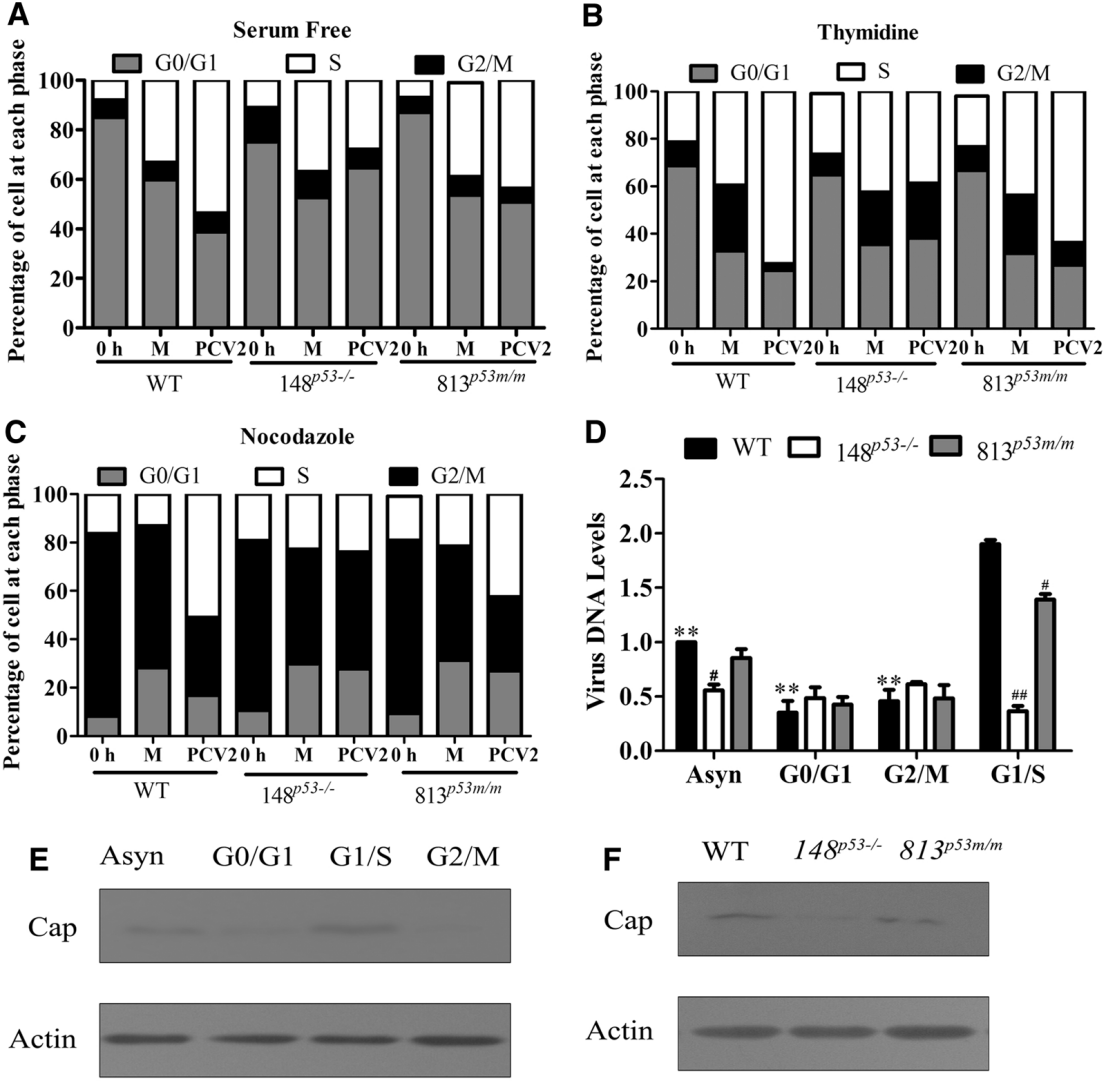
**S phase accumulation promotes PCV2 replication.** **A** PK15 cells, 148PK15^*P53*−*/*−^ cells, or 813PK15^*P53m/m*^ cells were cultured without serum for 48 h to synchronize at G0/G1 phase, followed by PCV2 (MOI = 1) or mock-infection. At 18 h post-infection, cells were stained with propidium iodide and applied to flow cytometry analysis. **B** Cells were synchronized in G1/S phase by 2 mM double-thymidine block for 12 h to release into the S phase. Then the cells were infected with 1 MOI PCV2 for 18 h and applied to flow cytometry analysis. **C** Cells were synchronized in G2/M phase by treatment with 100 ng/mL of nocodazole for 16 h. Then the cells were infected with 1 MOI PCV2 for 18 h and applied to flow cytometry analysis. **D** PCV2 ORF1 DNA levels were determined by qPCR at 18 h after infection (MOI = 1) in the cells. The data are mean ± SEM of three independent experiments. **p < 0.01 versus WT cells released from G1/S phase group. ^#^ *p* < 0.05, ^##^ *p* < 0.01 versus WT cells released from the same phase. **E** Western blot analysis of PCV2 Cap levels in the WT cells released from different synchronization treatments as above. **F** Western blot analysis of PCV2 Cap levels in WT PK15 cells, 148PK15^*P53*−*/*−^ cells, or 813PK15^*P53m/m*^ cells released from G1/S phase synchronized cells. Data represent three independent experiments.

To investigate the effects of S phase accumulation on PCV2 replication, we synchronized the cells at G0/G1 phase, G2/M phase and G1/S phase, and the cells were either mock infected or PCV2 infected. Then we analyzed the cell cycle profile as well as the PCV2 genomic DNA level and Cap protein level. The results show that using serum deprivation treatments for 48 h, over 80% of WT PK-15 and 75% of 148PK15^*P53*−*/*−^ cells were synchronized at the G0/G1 phase (Figure 5A). Using double-thymidine treatment, approximately 75% of WT cells and 70% of 148PK15^*P53*−*/*−^ cells were synchronized at the G1/S phase border (Figure 5B). Using nocodazole treatments, over 75% of WT PK-15 cells and 70% of 148PK15^*P53*−*/*−^ cells were synchronized at the G2/M phase (Figure 5C). In the WT cells released from the G0/G1 phase synchronized cells, the proportion of S phase cells increased about 23% in PCV2-infected cells compared to that in mock-infected cells at 18 h pi (Figure 5A). In the WT cells released from the G1/S synchronized cells, the cell population at the S phase increased about 38% in PCV2-infected cells compared to that in mock-infected cells at 18 h pi (Figure 5B). In the WT cells released from the G2/M phase synchronized cells, the cell population at the S phase increased about 40% in PCV2-infected cells compared to that in mock-infected cells at 18 h pi (Figure 5C). However, in 148PK15^*P53*−*/*−^ cells released from the G0/G1 phase, G1/S phase or G2/M phase synchronized cells, the cell population at the S phase in PCV2-infected cells decreased compared with that in wild-type cells or similar to that found in mock-infected cells at 18 h pi (Figure 5A–C). In 813PK15^*P53m/m*^ cells released from the G0/G1 phase, G1/S phase or G2/M phase synchronized cells, the cell populations at the S phase were also increased in PCV2-infected cells compared to those in mock-infected cells at 18 h pi, but which were also lower than those in PCV2-infected WT cells (Figure 5A–C).

Real-time PCR analysis of the genomic DNA of PCV2 shows that the replication levels of ORF1 DNA were higher in the WT cells released from G1/S-synchronized cells than those in the WT cells released from G0/G1 phase or G2/M phase synchronized, or asynchronous WT cells (Figure 5D). In addition, the levels of the PCV2 Cap protein also were higher in the WT cells released from G1/S-synchronized cells than those in the WT cells released from G0/G1 phase or G2/M phase synchronized or asynchronous cells (Figure 5E). However, the viral DNA levels and Cap protein levels were lower in the 148PK15^*P53*−*/*−^ and 813PK15^*P53m/m*^ cells released from the G1/S phase synchronized cells than those in the WT cells released from the G1/S phase synchronized cells (Figure 5D and F). These results suggest that PCV2 induction of S phase accumulation via p53 signaling might be beneficial for virus replication.

## Discussion

Viruses evolve complex strategies to override cell cycle checkpoints forcing host cells into specific phases to support viral replication. Some viruses interfere with the p53 surveillance pathways to promote replication [16, 17], other viruses exhibited a p53-independent way for its replication [18, 19]. In this study, we focused on whether the p53 signaling pathway contributes to PCV2 induced cell cycle progression and virus replication. The results show that PCV2 infection activated the p53 signaling to up-regulate p21 and Cyclin E while down-regulating Cyclin A and CDK2, forcing the infected cells to stay in the replicative S phase. The roles of p53 in PCV2 replication were further tested by infection of *p53* knockout and *p*53 mutant cells, in which the progression of the cell cycle was less effected by PCV2 and the replication of virus appears a relative lower level.

In this study, the first question we answered is whether PCV2 overrides cell cycle checkpoints for its benefits. In both asynchronic and synchronic cells, PCV2 induced cell cycle arrest at the S phase and promoted both PCV2 capsid protein and PCV2 progeny production, whereas UV-inactivated PCV2 did not exhibit S-phase accumulation. Besides this, PCV2 genomic DNA and Cap protein levels were higher in the WT cells released from G1/S-synchronized cells than those in the WT cells released from G0/G1 phase or G2/M phase synchronized, or asynchronous WT cells, suggesting that S phase accumulation might be beneficial for virus replication. Our data explain and agree with the previous reports that PCV2 DNA synthesis dramatically increases in PK15 cells right before mitosis [20], while the cell cycle regulator protein Cyclin A overexpression suppressed PCV2 replication [21]. Thus, we confirmed that PCV2 infection induced S-phase accumulation for its multiplication.

The other main question we answered is the roles of p53 signaling in modulating cell cycle arrest and PCV2 replication. The function of p53 in regulating the cell cycle progress of PCV2-infected cells was explored by comparing *p53* wild-type, knockout and mutant cell lines. Following PCV2 infection, p53 knockout caused significant reduction of DNA synthesis compared with the wild-type cell, whereas the p53 knockout cells exhibited a relatively stabilized high DNA synthesis rate whether in the presence or absence of PCV2 in the BrdU assay. We also observed that PCV2 genomic DNA levels and Cap protein levels were lower in the 148PK15^*P53*−*/*−^ and 813PK15^*P53m/m*^ cells released from G1/S phase synchronized cells than those in the WT cells released from G1/S phase synchronized cells. These results suggest that p53 plays a pivotal role in inducing S-phase cell accumulation and viral replication in PCV2-infected cells.

Actually, the role of p53 interplays between the host and virus has been extensively studied. Some viruses hijack the p53 apoptotic pathway to facilitate virus invasion, like Influenza A virus [22], Reovirus [23], and Epstein–Barr virus [24] while other virus proteins directly interact with p53 effecting both p53 DNA-binding affinity and transcriptional ability, such as Hepatitis C virus [25], and Hepatitis B virus [26]. In addition, some viruses specifically interrupt the cellular p53–p21 pathway to modulate the host cell cycle, blocking cellular DNA synthesis in generating viral nucleotide pools, like Respiratory syncytial virus [27], Herpes simplex virus type 2 (HSV-2), and Simian virus 40 (SV40). However, unlike HSV-2, which increases p21 protein levels by phosphorylation of the p53 protein at Ser20 [8], SV40 induced p53 phosphorylation is accompanied by Ser15 [28], as SV40 activating the ATR-Delta p53 signaling to maintain S phase environment, and to manipulate polymerases [17]. This may explain our data that in the CRISPR/Cas 9 mediated 148PK15^*P53*−*/*−^ cells, PCV2 infection did not induce S phase accumulation, resulting in a relative lower virus production, while the 813PK15^*P53m/m*^ cells that mutated original S271 and G272 sites of porcine p53 into R271 showed a slightly weakened function of p53 signaling undergoing PCV2 infection, resulting in a slight relative decreased S phase accumulation compared with the wild-type cell. Single amino acid mutation of p53 exhibited a different function, the human R273 site mutation induced a resistance to drug which induced apoptosis, while the mouse R270 residue is subject to stress-induced modifications [29, 30]. Similarly, in this study, the deletion of a single amino acid located at the sensitive key site, affected the progression of cell cycle in 813PK15^*P53m/m*^ cells. Interestingly, the delta p53 signaling, an alternative p53 isoform deleted from 256 to 322 amino acid residues, is activated upon the DNA virus SV40 infection or when the host cells encounter a single-strand DNA break, resulting in up-regulation of p21 and down-regulation of Cyclin A-CDK2 in S phase [17, 31]. In the current study, following PCV2 infection, an increase of p21 and a decrease of Cyclin A-CDK2 was observed, while in the p53 deficient cells, the level of expression of Cyclin A-CDK2 was efficiently altered to the basal level.

The CRISPR/Cas9-mediated gene editing system has emerged as a powerful and efficient tool to manipulate the genomes of potential targets to understand pathogeneses [32, 33]. Taking advantage of this technology allowed us to conduct basic research on p53 signaling in porcine cell lines. Even though the exact molecular mechanism of PCV2 infected cell response to p53 signaling still needs to be further studied, our data presented here confirmed that PCV2 induced cell cycle accumulates at the S phase for its replication, and p53 signaling contributes to this modulation process.

## Abbreviations

PCV2: porcine circovirus type 2
PCVAD: porcine circovirus associated diseases
KSHV: Kaposi’s sarcoma herpesvirus
PFV: prototype foamy virus
PK15: porcine kidney 15
PI: propidium iodide
MOI: multiplicity of infection
pi: post-infection
HSV-2: Herpes simplex virus type 2
SV40: Simian virus 40
